# Construction of *Brassica* A and C genome-based ordered pan-transcriptomes for use in rapeseed genomic research

**DOI:** 10.1016/j.dib.2015.06.016

**Published:** 2015-07-02

**Authors:** Zhesi He, Feng Cheng, Yi Li, Xiaowu Wang, Isobel A.P. Parkin, Boulos Chalhoub, Shengyi Liu, Ian Bancroft

**Affiliations:** a Department of Biology, University of York, Heslington, York YO10 5DD, UK; b Institute of Vegetables and Flowers, Chinese Academy of Agricultural Sciences, Beijing 100081, China; c Agriculture and Agri-Food Canada, 107 Science Place, Saskatoon, Canada S7N0X2; d URGV (Institut National de la Recherche Agronomique, Université Evry Val d’Essonne), Evry, France; e Oil Crops Research Institute, CAAS, No. 2 Xudong Second Road, Wuhan, Hubei, China

## Abstract

This data article reports the establishment of the first pan-transcriptome resources for the *Brassica* A and C genomes. These were developed using existing coding DNA sequence (CDS) gene models from the now-published *Brassica oleracea* TO1000 and *Brassica napus* Darmor-*bzh* genome sequence assemblies representing the chromosomes of these species, along with preliminary CDS models from an updated *Brassica rapa* Chiifu genome sequence assembly. The *B. rapa* genome sequence scaffolds required splitting and re-ordering to match the expected genome organisation based on a high density SNP linkage map, but the *B. oleracea* assembly was used unchanged. The resulting *B. rapa* (A genome) pseudomolecules contained 47,656 ordered CDS models and the *B. oleracea* (C genome) pseudomolecules contained 54,766 ordered CDS models. Interpolation of *B. napus* CDS models not already represented by orthologues resulted in 52,790 and 63,308 ordered CDS models in the A and C pan-transcriptomes, an increase of 13,676 overall. Comparison of the organisation of this resource with publicly available genome sequences for *B. napus* showed excellent consistency for the *B. napus* Darmor-*bzh* resource, but more breakdown of collinearity for the *B. napus* ZS11 resource. CDS datasets comprising the pan-transcriptomes are available with this article (*B. rapa*) or from public repositories (*B. oleracea* and *B. napus*).

 Specifications Table

**Table:** 

Subject area	Biology
More specific subject area	Plant genome organisation
Type of data	CDS gene model sequences for the A genome, in FASTA format. Tables (in the form of MS Excel spreadsheets) providing A genome pseudomolecule specification based on genome sequence scaffolds, inferred order and anchoring positions in the A and C genome pseudomolecules for CDS models and a figure illustrating the collinearity of the ordered pan-transcriptome and two genome sequences reported for *B. napus*.
How data was acquired	CDS gene model sequences for the A genome were developed as part of the reported work. Genome sequence scaffolds and other CDS data were obtained from the groups generating them prior to publication.
Data format	The data accompanying this article are provided as text files (for *B. rapa* CDS models and R scripts) and MS Excel spreadsheets providing CDS and scaffold identifiers and sequence similarity coordinates.
Experimental factors	n/a
Experimental features	CDS modelling was undertaken using V2.0 *B. rapa* genome sequence scaffolds. A previously-reported set of *Brassica* A genome pseudomolecules was used to produce improved pseudomolecules derived from an updated B. rapa genome assembly in order to represent the organisation of the A genome in *B. napus*. Integration and interpolation of gene models called only in a *B. napus* genome sequence was undertaken, resulting in the establishment of a pan-transcriptome resource for the *Brassica* A and C genomes. Collinearity analysis with public *B. napus* genome sequences was undertaken, based on BLAST similarity hits of CDS models, to compare the order of genes in the pan-transcriptome resource with that of their orthologues in two published *B. napus* genome sequences.
Data source location	SRA, NCBI, ENA
Data accessibility	All genome sequence datasets were provided for analysis prior to publications, but are now available:
	The *B. napus* Darmor-*bzh* assembly is available at ENA (European Nucleotide Archive), in the WGS section for contigs (accession numbers CCCW010000001 to CCCW010044187) and the CON section for scaffolds, chromosomes, and annotation (accession numbers LK031787 to LK052685). The *B. napus* ZS11 assembly is available at http://www.ncbi.nlm.nih.gov/Traces/wgs/?val=JMKK01#. The *B. oleracea* assembly is available via Sequence Read Archive accession number PRJNA158027. The *B. rapa* version 2 assembly is in the process of publication and in the meantime is available from Xiaowu Wang (wangxiaowu@caas.cn).

**Value of the data**

• Provides an updated pseudomolecule description, with genome sequence scaffolds from *B. rapa* and *B. oleracea* representing genome organisation in *B. napus*
• Provides for the first time pan-transcriptome resources for use in *Brassica* species containing the A and/or C genomes
• Provides insights into the extent of gene content variation between the *Brassica* A and C genomes as represented in an allopolyploid and its diploid progenitors
• Provides a hypothetical gene order resource for the *Brassica* A and C pan-genomes for use in genome evolution studies and Associative Transcriptomics.

## 1 Experimental design, materials and methods

Transcriptome-based molecular marker systems have been developed and deployed with great success in the crop species *B. napus* for both genome organisation studies [1] and association genetics [2]. These studies exploit mRNAseq data, which need to be mapped to a suitable transcriptome reference sequence for single nucleotide polymorphism (SNP) identification and transcript quantification. The first generation approach used unigene assemblies as the reference sequences [3], which permitted some resolution of the contributions to the transcriptome of homoeologous gene pairs [4]. However, the genome sequences reported for *B. napus* Darmor-*bzh* indicate that sequence exchanges between the constituent genomes of this allotetraploid species (A genome from an unknown *B. rapa* and C genome from an unknown *B. oleacea*) may occur very frequently [5], making it imperative that the genome-of-origin of any given gene be determined as clearly as possible. The most reliable way of achieving this is to base resources primarily on those derived from the constituent genomes in the diploid progenitors of *B. napus*, *i.e.* from *B. rapa* and *B. oleracea*. As an improvement on the existing resource based on unigenes assembled across *Brassica* species [3,6], we therefore aimed to develop a new transcriptome reference, based on coding DNA sequence (CDS) gene models derived primarily from the *Brassica* A and C genomes as represented in the progenitor species. As the *B. napus* genome sequence annotation identified many gene models without orthologues in *B. rapa* and *B. oleracea*, we further aimed to interpolate those *B. napus*-specific CDS models, thus producing pan-transcriptome resources for the *Brassica* A and C genomes as represented by the union of orthologous genes of *B. rapa*, *B. oleracea* and *B. napus*.

The version 2 *B. rapa* Chiifu genome sequence scaffolds represent a major advance on the published version 1 sequences [7] in that they provide more comprehensive coverage of the genome, with aggregate scaffold size increasing from 248 Mb to 370 Mb. A preliminary annotation was undertaken of the genome sequence scaffolds that had been organised into chromosomes, essentially as described for the version 1 genome sequences [7]. Briefly: Genscan and Augustus with parameters established using *Arabidopsis thaliana* gene models were used to perform *de novo* gene predictions in the new genome assembly of *B. rapa*, after masking the Class I and Class II transposable elements. The predicted genes with CDS models shorter than 150 bp were filtered out. We further performed homology based gene prediction by aligning *A. thaliana*, *Carica papaya*, *Populus trichocarpa*, *Vitis vinifera* and *Oryza sativa* protein sequences to the *B. rapa* genome. TBLASTN was used to do fast alignment (threshold *e*-value 1E−5), then Genewise was used to do precise alignment. Additionally, we assembled the *Brassica* ESTs downloaded from NCBI using PASA and aligned them to *B. rapa* genome by BLAT. Considering that the fragmented exons in EST data might lead to false results, we filtered out alignments with gaps (introns) that span over 10 kb in length. We then ran GLEAN to merge the gene sets generated from *de novo* and homology-based predictions, using mRNA-Seq data as the supporting evidence. Finally, the *B. rapa* gene set was aligned to the TE protein database of Repbase, those hits with *e*-value>1E−5 and coverage≥50% were filtered out. The remaining gene models were reported as *Brassica* gene set Version 2.0 (Additional file 1). These CDS models were then used in sequence similarity searches using BLAST to identify the highest-scoring significant hit (threshold *e*-value 1E−30) for each CDS model in both the version 2 *B. rapa* Chiifu genome sequence scaffolds and the A genome pseudomolecules reported previously [6], based on the version 1 *B. rapa* Chiifu genome sequence [7] that had been reordered relative to the *B. napus* genome via high density transcriptome SNP linkage mapping [1]. This enabled the identification of chimeric scaffolds in the version 2 assembly that could be split (Additional file 2) and re-organised (Additional file 3) to form pseudomolecules representative of the organisation of the *Brassica* A genome. The CDS models from the *B. oleracea* TO1000 [8] genome sequence were similarly used to assess collinearity with the C genome pseudomolecules reported previously [6] and were found to be in excellent agreement, so the *B. oleracea* TO1000 assembly was adopted unaltered as representing the *Brassica* C genome pseudomolecule resource.

The *B. rapa* Chiifu CDS, along with CDS from the published *B. oleracea* TO1000 genome sequence [8], was mapped onto the respective genome sequence pseudomolecules using BLAST to identify the highest-scoring significant hit (threshold *e*-value 1E−30). This resulted in the mapping and ordering of 47,656 *B. rapa* CDS models to the A genome and 54,766 *B. oleracea* CDS models to the C genome. A total of 101,040 CDS models were annotated in the *B. napus* Darmor-*bzh* genome [5]. Of these, 80,927 CDS models which had been anchored to the 19 *B. napus* pseudomolecules were mapped onto the respective (*B. rapa* and *B. oleracea*-based) genome sequence pseudomolecules by BLAST (threshold *e*-value 1E−30). *B. napus* CDS models mapping redundantly with CDS models derived from *B. rapa* and *B. oleracea* (threshold *e*-value 1E−30) were excluded, resulting in the addition of 2165 and 3032 CDS models to the A and C genomes, respectively. Finally, CDS models from the *B. napus* Darmor-*bzh* genome sequence that did not have significant (threshold *e*-value 1E−30) BLAST hits in the (*B. rapa* and *B. oleracea*-based) genome sequence pseudomolecules were interpolated based on the positions of flanking gene models that did map. This was done by combining the *B. napus* Darmor-*bzh* CDS models׳ sorted location on the *B. napus* Darmor-*bzh* chromosome with the mapped location of flanking genes on the *B. rapa* or *B. oleracea*-based pseudomolecules using an R script (Additional file 4) to perform the following: (1) Sort *B. napus* CDS models by *B. napus* Darmor-*bzh* pseudomolecules, then by their *B. rapa* or *B. oleracea-*based pseudomolecules hit locations then (2) CDS models (or runs of adjacent CDS models) that do not have a hit onto the *B. rapa* or *B. oleracea-*based pseudomolecules are interpolated onto those pseudomolecules with a three digit suffix starting from the boundary of the point of insertion. When the boundaries are not in the right order, the interpolation starts from the closest boundary number to the mean of the nearest 10 neighbours of the run of CDS models. If there is no mapping in the 10 nearest neighbours, the interpolation starts from the minimum of the boundary numbers. This resulted in the addition of 2969 and 5510 further CDS models to the A and C genomes, respectively. The final AC pan-transcriptome resource therefore comprises a total of 116,098 hypothetically ordered CDS models (Additional file 5,6,7), 52,790 in the *Brassica* A genome and 63,308 in the *Brassica* C genome. This represents an increase of 35,171 over the 80,927 CDS models annotated in the published *B. napus* Darmor-*bzh* pseudomolecules, 15,058 over the complement of gene models for *B. napus* including the 20,113 in sequence scaffolds not incorporated into the *B. napus* Darmor-*bzh* pseudomolecules [5] and 13,676 more than had been identified in the *B. rapa* and *B. oleracea* pseudomolecules.

**Fig. 1:**
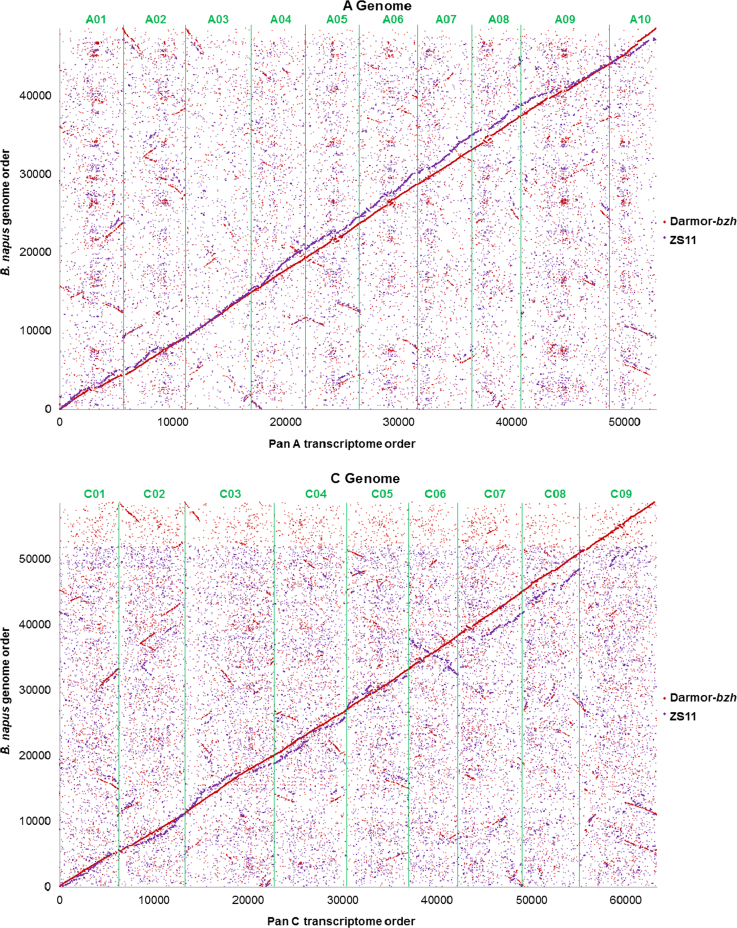
Collinearity of ordered pan-transcriptomes and the genome sequences of *B. napus* Darmor-*bzh* and *B. napus* ZS11. The positions of best sequence matches in the *B. napus* chromosome assemblies are plotted for CDS models with significant similarity matches (threshold *e*-value 1E−30) in the *B. napus* Darmor-*bzh* assembly and *B. napus* ZS11 assembly.

The order of CDS models in the pan transcriptome was compared with the order of orthologous sequences in two publicly-available *B. napus* genome sequence resources. This was conducted by sequence similarity search using BLAST to identify the highest-scoring significant hit (threshold *e*-value 1E−30) for each CDS model in the pan-transcriptome in each of the *B. napus* Darmor-*bzh* and *B. napus* ZS11 chromosome assemblies. Of the aggregate 116,098 CDS models in the pan-transcriptome, 107,292 (92.4%) returned significant hits (threshold *e*-value 1E−30) in the *B. napus* Darmor-*bzh* assembly and 99,395 (85.6%) returned significant hits in the *B. napus* ZS11 assembly. The order of these best similarity matches in each resource is illustrated in Fig. 1. The inferred gene order in the pan-transcriptome and the *B. napus* Darmor-*bzh* genome assembly shows excellent collinearity. A small number of local rearrangements can be observed in regions with relative high densities of non-collinear matches, possibly corresponding to paracentromeric regions. In addition, two prominent segments shadowing the main collinearity diagonal can be observed amongst the background of CDS models mapping to non-orthologous positions. Such shadows have been observed in previous studies [6] and were shown to correspond to sequences missing from the genome sequence resource, with consequent mapping of sequences to one of the two paralogous segments of these paleohexaploid genomes. The inferred gene order in the pan-transcriptome and the *B. napus* ZS11 genome assembly show extensive collinearity, but with more disruption by rearrangements than was observed with *B. napus* Darmor-*bzh* resource. Linkage group C6 is also presented in the *B. napus* ZS11 genome sequence resource in the opposite orientation to the current reference genetic map for the *Brassica* C genome. These analyses, which together indicate extensive collinearity of the *Brassica* A and C genomes as represented in the allotetraploid *B. napus* and representatives of its progenitors, are also consistent with early observations of extensive collinearity, but with some divergence in gene content between orthologous regions of *Brassica* genomes, including both loss and mobility of coding sequences [9].
